# Developing a prognostic signature and characterizing the tumor microenvironment based on centrosome-related genes in lung adenocarcinoma

**DOI:** 10.32604/or.2025.056176

**Published:** 2025-06-26

**Authors:** LINGJIE XU, YIQIN XIA, QIN QIN, GUIQUN WANG, KAI TAO, WEI WEI

**Affiliations:** 1 Department of Emergency, West China Hospital, Sichuan University, Chengdu, 610041, China; 2 West China School of Medicine, Sichuan University, Chengdu, 610041, China

**Keywords:** Centrosome, Lung adenocarcinoma, Neoplastic microenvironment, Personalized treatment, Prognostic model

## Abstract

**Background:**

The centrosome, a crucial cellular structure involved in the mitotic process of eukaryotic cells, plays a significant role in tumor progression by regulating the growth and differentiation of neoplastic cells. This makes the centrosome a promising target for therapeutic strategies in cancer treatment.

**Methods:**

Utilizing data from the TCGA database, we identified centrosome-related genes and constructed a prognostic model for 518 lung adenocarcinoma patients. Prognosis-associated genes were initially screened using univariate Cox regression, with overfitting minimized by applying LASSO regression to remove collinearity. Finally, a set of 12 genes was selected through multivariable Cox regression for inclusion in the prognostic model.

**Results:**

The model’s performance was assessed using ROC curve analysis, demonstrating a robust predictive ability with an AUC of 0.728 in the training group and 0.695 in the validation group. Differential expression analysis between high-risk (HRLAs) and low-risk (LRLAs) individuals was performed, followed by enrichment analyses using KEGG, GO, Progeny, GSVA, and GSEA. These analyses revealed significant differences in immune-related pathways between the two groups. Immune microenvironment assessment through ssGSEA and ESTIMATE indicated that individuals with poor prognosis exhibited lower immune, stromal, and ESTIMATE scores, along with higher tumor purity, suggesting an impaired immune microenvironment in HRLAs patients. Drug susceptibility analysis and molecular docking showed that HRLAs individuals were more responsive to docetaxel, emphasizing the therapeutic relevance of paclitaxel in this cohort.

**Conclusion:**

We successfully developed and validated a centrosome-associated gene-based prognostic model, offering clinicians valuable insights for improved decision-making and personalized treatment strategies. This model may facilitate the identification of high-risk patients and guide therapeutic interventions in lung adenocarcinoma.

## Introduction

Lung adenocarcinoma is a malignant tumor that typically originates in the lungs or respiratory tract, and is classified as a subtype of lung cancer [1]. It is one of the most common forms of lung cancer, primarily affecting middle-aged and elderly individuals, though it can occur at any age [2]. Symptoms often include coughing, sputum production, shortness of breath, chest pain, and hemoptysis [3]. Treatment options for lung adenocarcinoma generally include surgical tumor removal, chemotherapy, and radiation therapy [4]. These strategies are customized based on factors such as tumor stage, size, location, and the patient’s overall health [5]. Surgery is typically the first line of treatment, while chemotherapy is used to prevent recurrence and manage tumor growth [6]. Radiation therapy is also effective in eradicating any remaining cancer cells [7].

Recently, the treatment of lung adenocarcinoma has expanded to include targeted therapy and immunotherapy, which aim to improve survival and recovery rates [8]. The prognosis of lung adenocarcinoma is affected by factors such as the stage of the disease, tumor size and location, degree of differentiation, the patient’s overall health, and the chosen treatment approach [9]. Early detection and timely intervention can significantly improve outcomes, enhancing recovery and extending life expectancy. Patients diagnosed at an early stage and treated promptly tend to have a higher 5-year survival rate compared to those diagnosed and treated at a more advanced stage [10].

The centrosome is a cellular organelle composed of multiple proteins and RNA, located near the nucleus, and is involved in several critical biological processes, including signal transduction, cell polarity, and cell cycle regulation [11]. Recent research has increasingly emphasized the crucial role of the centrosome in cancer initiation and progression [12]. Certain DNA repair proteins, which become dysregulated and contribute to tumor development, are localized at the centrosomes [13,14]. In many cancer cells, centrosomes undergo notable changes in number, size, shape, and composition. One prominent alteration is centrosome amplification, which leads to abnormal spindle formation during cell division, resulting in genomic instability. In terms of size, centrosomes in cancer cells are often enlarged, possibly due to elevated levels of microtubule-associated proteins, which interfere with normal cell division. The shape of centrosomes may also become irregular, such as distorted spherical or elliptical forms, indicating functional abnormalities during mitosis. Additionally, cancer cells may exhibit abnormal expression of centrosomal proteins, which disrupts the assembly of spindle microtubules and causes inaccurate chromosome segregation, further promoting genomic instability. These changes collectively contribute to the rapid proliferation and aggressive behavior of cancer cells, making centrosome abnormalities a defining feature of cancer. Research has demonstrated that centrosomes play a pivotal role in several cellular processes that drive cancer progression, including the regulation of cell division, proper chromosome segregation, and the maintenance of genomic stability. Consequently, abnormalities in centrosomes can contribute to various oncogenic processes, such as uncontrolled proliferation, metastasis, drug resistance, and immune evasion. Moreover, many proteins and RNAs associated with the centrosome exhibit dysregulated expression or genetic alterations in cancer, underscoring the centrosome’s critical role in modulating these processes.

As a result, researchers now regard the centrosome as a critical target for cancer therapies and are developing innovative drugs and treatment strategies aimed at it. Approaches such as RNA interference, monoclonal antibodies, and small-molecule agents targeting centrosome-associated proteins and RNA have been employed in cancer treatments, showing promising early results [15]. Additionally, certain centrosome proteins have been identified as prognostic markers for cancer, aiding clinicians in evaluating treatment effectiveness and predicting overall patient outcomes [16].

The immunological microenvironment consists of a diverse array of elements surrounding a tumor, including cancer cells, immune cells, endothelial cells, fibroblasts, and various signaling molecules [17]. These components interact in complex ways, collectively influencing tumor growth, metastasis, and response to treatment. Cancer heterogeneity and drug resistance further complicate these interactions, making it difficult to develop universally effective therapeutic strategies. Understanding how the immunological microenvironment contributes to these challenges is crucial for improving treatment outcomes. Immunotherapy, which employs immune cells and molecules such as immune checkpoint inhibitors, CAR-T cell therapy, and vaccines, has emerged as a promising approach to cancer treatment [18]. Recent research has increasingly focused on the immune microenvironment and its role in immunotherapy. Numerous studies have demonstrated that the immunological environment plays a critical role in tumor formation, progression, and therapeutic response. By altering the components and functions of the immune microenvironment, tumor cells can evade immune detection, thereby diminishing the effectiveness of immunotherapy [19]. On the other hand, immunotherapy has shown remarkable success in certain cancers, such as melanoma, osteosarcoma, and breast cancer [20–22]. Growing evidence suggests that the efficacy of immunotherapy is closely tied to the condition of the immune microenvironment, with the degree of immune cell infiltration in certain tumors correlating with better treatment outcomes [23]. Therefore, understanding the relationship between the immune microenvironment and immunotherapy could lead to more effective cancer treatment strategies.

However, there is a lack of research on the impact of the centrosome on the immune microenvironment and prognosis in lung adenocarcinoma. This study aimed to explore the roles of centrosome-related genes in lung adenocarcinoma. Additionally, we developed a predictive model to identify potential biomarkers and therapeutic targets for personalized immunotherapy based on this information. This model is designed to predict patient prognosis and assist in guiding clinical treatment decisions.

## Materials and Methods

### Obtaining and cleaning data

We acquired RNA sequencing data, along with survival details, clinical characteristics, and single nucleotide variations for lung adenocarcinoma patients from the TCGA (The Cancer Genome Atlas) database. Due to the incomplete uniformity of information contained for each patient, we retained only those patients who had specific sets of information for the corresponding analysis. Initially available in count format, the RNA-seq data was transformed to log2(TPM+1) for more accurate assessment of gene expression levels. Additionally, based on prior studies, we identified 726 centrosome-associated genes (CAGs) for our analyses [24].

### DEGs filter

We conducted differential gene expression analysis comparing adjacent normal tissue with tumor tissue, and between high-risk LUADs (HRLAs) and low-risk LUADs (LRLAs) groups. For these analyses, we used RNA-seq data in count format and processed the results with the DESeq2 package (Version 1.40.2). For each gene, we determined the log2 fold change between groups and calculated the adjusted *p-value*. Genes showing an absolute log2 fold change greater than 1 and an adjusted *p-value* below 0.01 were considered differentially expressed between the groups. We then visualized the results with a heatmap and volcano plot to depict the expression levels and regulation patterns of differentially expressed genes.

### Assessing CAGs-based prognostic signatures

After integrating the transcriptome profile and survival data of LUAD sufferers, we divided them into a train group and a validation group in a 7:3 proportion. We developed a model using the train group and evaluated its performance on the validation group. First, we performed a univariate Cox regression to distinguish CAGs linked to prognosis. Next, we applied LASSO regression to address collinearity and subsequently built a prognostic model with multivariate Cox regression. We carried out these procedures using the ‘glmnet’ package in R. This model allows us to assess patient prognosis with CAGs model score (CAGS) for each sample. The CAGS can be calculated using the following formula:

$$\begin{array}{rl} CAGS= & \sum_{i=1}^{n}regression\;coefficient\;of\;gene\;i\\ & \times expression\;of\;gene\;i \end{array}$$

We then evaluated CAGS’s accuracy on both groups using ROC curves, created using the ‘survivalROC’ package in R. Finally, we used CAGS to classify the sufferers into HRLAs and LRLAs categories.

### A nomogram was created to predict overall survival duration

After developing the prognostic model based on centrosome-related genes, we used the ‘rms’ package to create nomograms. These nomograms offer an easy tool for predicting patient prognosis by incorporating factors like sex, age, tumor stage, and risk category within the model. We then used temporal calibration curves to evaluate the performance of our nomograms.

### Tumor mutation landscape

We utilized the ‘maftools’ package to examine the gene mutation status in tumors and visualized the findings with waterfall plots. We selected genes with a high mutation rate in the tumor as well as our model genes for presentation. Next, we calculated the tumor mutation burden and compared the differences between the HRLAs and LRLAs.

### Functional analysis derived from differentially expressed CAGs

As described in the above-mentioned methods, after conducting two differential gene expression analyses, we obtained two lists of differentially expressed CAGs. To elucidate the sence of these genes, we conducted enrichment analysis using the GO (Gene Ontology Database) and KEGG (Kyoto Encyclopedia of Genes and Genomes) databases. We applied a filter with an FDR cutoff of 0.05 to refine our results and utilized the ‘enrichplot’ (Version 1.20.3) and ‘ggplot2’ (Version 0.4.9) packages in R for visualization.

### Analyses using GSVA and GSEA

GSVA (Gene Set Variation Analysis) and GSEA (Gene Set Enrichment Analysis) are two commonly applied methods for analyzing genomic data, specifically for studying the functionality and pathways of gene sets. GSVA is used to assess the activity of gene sets and is an unsupervised method that primarily focuses on the relative abundance of gene sets within samples. It evaluates the activity of biological processes in individual samples by considering the expression profiles of all genes in a single patient. GSEA is applied to identify the enrichment status of gene sets and is a supervised method that primarily focuses on the expression changes of gene sets under different experimental conditions. In our study, we calculated the logFC values for all genes between the HRLAs and LRLAs, ranked them, then performed GSEA evaluation to assess the biological functional differences of upregulated and downregulated genes. Subsequently, we applied the ‘progeny’ package to investigate the activity of 14 classic tumor-associated pathways, which essentially involves the use of the GSVA method. We utilized KEGG and GO standard gene sets for GSVA and GSEA analyses, respectively, to assess pathway scores and gene enrichment between the HRLAs and LRLAs. This approach provided a clear understanding of differences of biological processes within the two patient groups.

### Assessing immune infiltration and profiling the immune microenvironment

There are diverse algorithms available to assess immunity-related cell infiltration within samples based on transcriptomic data. The methods can be categorized into marker-based methods and deconvolution-based methods. Certain methods facilitate intra-sample comparisons between different cell types, while others permit inter-sample comparisons for the same cell types. In this study, we employed six immune infiltration algorithms to assess the immune microenvironment of the samples, including EPIC, quantTIseq, CIBERSORT, XCELL, MCPcounter, and TIMER. Among them, EPIC and quantTIseq can provide the absolute content of immunological cells within the samples. Therefore, we displayed our results of these two algorithms in the main text figures, while the results of the other methods were presented in the supplementary materials. Next, we utilized the ESTIMATE package to compute four scores for the samples, including the overall immune score, tumor purity score, stromal score, and ESTIMATE score. Ultimately, we examined the variations in these scores between the HRLAs and LRLAs.

### Drug response

To evaluate the efficacy of medications and identify potential therapeutic aims, we utilized the ‘oncoPredict’ (Version 0.2) software package to conduct a sensitivity analysis on 198 drugs. We calculated the IC50 values for each drug within the samples. Following this, we compared the IC50 values between the two sample groups, selecting those medications with a mean IC50 value under 1 for additional examination.

### Immunohistochemistry from human protein atlas

We retrieved immunohistochemistry data for the model genes from both normal tissue and lung adenocarcinoma tissue using the HPA database (https://www.proteinatlas.org/ (accessed on 04 December 2024)). This enabled us to evaluate the variations in expression levels between the two tissue types, thereby confirming the findings from our earlier analyses.

### A549 cultivation

A549, a human lung carcinoma cell line, were procured from Procell (Wuhan, China). We detected the presence of mycoplasma contamination in the cells by PCR amplification assay to ensure that the cells used in the experiment were free of mycoplasma. The cells were cultured in RPMI-1640 medium (Gibco 11875093, Beijing, China), supplemented with 10% fetal bovine serum (FBS 10270106, Gibco, Beijing, China) and 1% penicillin-streptomycin (Gibco 15140122, Beijing, China). All cell culture activities were conducted in a humidified incubator at 37°C with 5% CO_2_. When cells reached about 80% confluency, they were detached using 0.25% trypsin-EDTA solution (Gibco 25200056, Beijing, China) and subsequently subcultured at a 1:3 ratio. Cell counts and viability were determined using the Trypan blue exclusion method, ensuring cell viability exceeded 95% before experiments. Before conducting experiments, A549 cells were seeded at a density of 1 × 10^5^ cells/mL in appropriate culture dishes and pre-incubated for 24 h to allow for cell attachment.

### Cell transfection

A549 were cultured in 6-well plates until they reached 70%–80% confluency. Transfection was carried out using Lipofectamine 2000 reagent (Invitrogen 11668019) and si-RNA (PRC1-3’UTR siRNA targeting sequence:

5′-CGCUGUUUACUCAUACAGU-3′, the sequence of nagetive control siRNA is 5′-UUCUCCGAACGUGUCACGU-3′), adhering to the manufacturer’s guidelines. The DNA-Lipofectamine 2000 complexes were formed in Opti-MEM I Reduced Serum Medium (Gibco 31985070, Beijing, China) and added to the cells after replacing the existing medium with fresh RPMI-1640 medium devoid of antibiotics. Post-incubation, the medium was changed to fresh RPMI-1640 supplemented with 10% FBS and 1% penicillin-streptomycin.

### Real-time quantitative PCR

Total RNA was isolated from A549 using the TRIzol (Beyotime R0016, Shanghai, China) reagent, following the instructions provided by the manufacturer. The RNA quality and concentration were measured with a NanoDrop spectrophotometer (Thermo Scientific NanoDrop, Shanghai, China). cDNA was synthesized utilizing the High-Capacity cDNA Reverse Transcription Kit (Thermo 4387406, Shnghai, China), with 1 µg of total RNA serving as the template. Real-time quantitative PCR was then performed using the SYBR Green PCR Master Mix (Serviebio G3322-01, Wuhan, China) on a QuantStudio 5 Real-Time PCR System (Bio-Rad CFX Connect, Shanghai, China). The primers used for human PRC1 and GAPDH are: PRC1 forward primer GCTGGAAGAAGAGTTGAAGG, PRC1 reverse primer CTTCAGTTGTCTTTTCCTGCT, GAPDH forward primer GTTCGTCACTGGGTGTGAACCA, and GAPDH reverse primer AGTCCTTCCACGATACCAAAGT.

### Western blot

A549 were lysed in RIPA buffer containing protease and phosphatase inhibitors (Servicebio G2002-100ML, Wuhan, China) to extract proteins. The protein concentration was then determined using a BCA protein assay kit (Beyotime P0012, Shanghai, China), according to the manufacturer’s instructions. Equivalent protein amounts were resolved by SDS-PAGE on a polyacrylamide gel and subsequently shifted to PVDF (Polyvinylidene fluoride, Cytiva 10600023, Shanghai, China) membrane. Avoiding non-specific combination, the membrane was blocked with 5% non-fat milk in TBST (Tris-buffered saline with 0.1% Tween 20). The membrane was then incubated with PRC1 primary antibodies (PRC1: Proteintech 15617-1-AP, Wuhan, China; GAPDH: Proteintech 10494-1-AP, Wuhan, China) specific to the target protein at a dilution of 1:3000 and GAPDH primary antibodies at a dilution of 1:10000, followed by incubation with HRP-conjugated secondary antibodies (Proteintch SA00001-2, Wuhan, China) at room temperature for 1 h. The primary antibody incubation was carried out overnight at 4°C on a shaker. Antibody incubations were conducted in blocking solution. Protein detection was achieved using an enhanced chemiluminescence (ECL) system (Bio-Rad ChemDoc Mp, Shanghai, China), and signals were captured either on X-ray film or a digital imaging system. Band intensity was quantified with image analysis software (ImageJ 1.54 g, Wayne Rasband and contributors, National Institutes of Health, Bethesda, MD, USA) to compare the relative expression levels of the target protein(s) across different samples.

### Cell proliferation assay using CCK-8

A549 cells were plated in 96-well plates at a density of 5000 cells per well to ensure they remained in exponential growth throughout the assay period. Following treatment with the test compounds or conditions for a designated time, the medium was changed with fresh medium containing the Cell Counting Kit-8 (CCK-8) (Beyotime C0037, Shanghai, China) solution, as per the manufacturer’s instructions. The plates were then incubated for an additional hour in a humidified incubator set at 37°C with 5% CO_2_. Absorbance was measured at 450 nm using a microplate reader (Thermo **A51119600C,** Shanghai, China), with the absorbance values directly correlating to the number of viable cells.

### Colony formation assay

For the Clonogenic assay, A549 were initially seeded in six-well plates with a density of 5 × 10^2^ cells per well. This step ensures that the number of cells in each well is low enough to allow individual cells to grow independently and form visible colonies. After seeding, the cells were cultured in an incubator for approximately 10 days. This period allows the cells sufficient time to divide and proliferate, resulting in colonies of various sizes.Upon completion of the culturing process, to observe and analyze these formed colonies, they need to be fixed and stained. Firstly, the colonies are fixed with 4% paraformaldehyde, a step crucial for preserving the integrity of the cell structure and preventing damage during subsequent processing. Following fixation, the colonies were colored with 0.1% crystal violet.

### Wound healing assay

Seed the treated A549 cells in a 6-well plate and, once fully grown, use the tip of a micropipette to scratch the monolayer, simulating a wound. Subsequently, replace the existing RPMI-1640 medium with serum-free medium to promote cell migration towards the wounded area. To monitor and evaluate cell migration, photographs of the wounded area are recorded at 0-, 24- and 48-h post-scratch employing an microscope (Olympus IX81, Japan). The area of the cell wound is quantitatively analyzed using ImageJ software (Version 1.54 g). The cell migration ability is assessed by computing the rate of wound curing areas at 24 and 48 h, employing the following method for calculation:

Healing area percentage = (area of 0–24 h or 48 h)/(area of 0 h)

### Transwell migration evaluation

Following a series of cell treatments, 4 × 10^4^ cells were suspended in serum-free media and seeded into the distal chamber of a transwell apparatus coated with matrigel (Beyotime C0371, Shanghai, China). This setup aims to mimic the extracellular matrix to better study cell invasiveness. The media in the down layer chamber contained 10% FBS, serving as a potent chemical attractant to encourage cells to migrate through the porous membrane. After 24 h of incubation, cells completed their migration from the distal to the down chamber. At this point, A549 that did not pass through the membrane were gently removed from the membrane’s upper surface. A549 that successfully migrated and passed the membrane to reach the lower chamber were fixed using pre-cooled paraformaldehyde to preserve their morphology. Subsequently, these cells were stained with crystal violet, a dye that binds to cellular DNA, making the cells easier to observe under a microscope. Finally, these fixed and stained cells were counted and photographed using an Olympus microscope.

### Statistical examination

The Wilcoxon rank-sum test is utilized to evaluate whether a significant difference exists between two distinct groups. Conversely, the Kruskal-Wallis test is applied to determine if a significant difference is present among three or more data groups. This statistical evaluation was conducted using the R package, version 4.0. Results achieving statistical significance were considered in all analyses, adopting a threshold of less than 0.05. Significance levels were denoted as follows: “*” for *p* < 0.05, “**” for *p* < 0.01, “***” for *p* < 0.001 and “****” for *p* < 0.0001. The number of repetitions for each assay (n) is indicated in the figure legends.

## Results

### Differences in CAGs between normal tissue and lung adenocarcinoma

**Figure 1:**
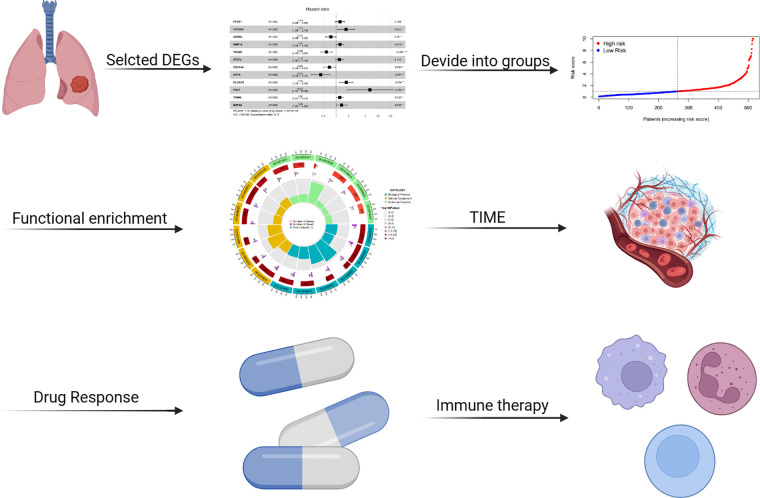
Overview of the analysis workflow.

**Figure 2:**
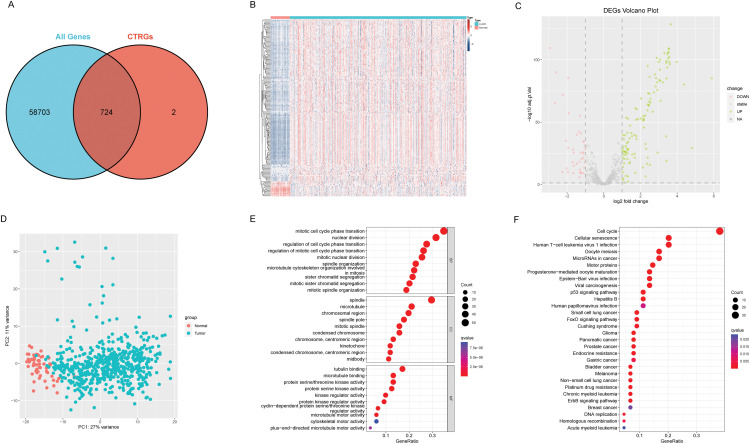
Differences between adjacent normal tissue and lung adenocarcinoma tissue. (A) Venn diagram illustrates the intersection between centrosome-related genes and the entire transcriptome. (B) The heatmap illustrates gene expression and differential patterns between the two sample groups. (C) The volcano plot displays upregulated and downregulated genes along with their respective fold changes. (D) The PCA (Principal Component Analysis) plot demonstrates significant separation between the two sample groups. (E) The bubble plot illustrates the results of GO enrichment analysis for differentially expressed genes. (F) The bubble plot illustrates the results of KEGG enrichment analysis for differentially expressed genes.

The complete analysis workflow is depicted in Fig. 1. Out of the 726 centrosome-associated genes (CAGs) analyzed, RNA-seq data were available for 724 genes (Fig. 2A). Among these, 115 genes exhibited significant differential expression between normal and tumor tissues (Fig. 2B), with 76 being upregulated and 39 downregulated in tumor tissues (Fig. 2C). Principal component analysis revealed a clear separation between normal and tumor samples (Fig. 2D). We then conducted functional enrichment analysis on the differentially expressed CAGs. In the Gene Ontology (GO) analysis, these genes were primarily associated with biological processes related to the cell cycle, such as mitotic cell cycle phase transition and sister chromatid segregation (Fig. 2E). In the KEGG pathway analysis, ‘cell cycle’ emerged as the most significantly enriched pathway, alongside other notable pathways including oocyte meiosis and tumor-related pathways (Fig. 2F). Based on these findings, we conclude that cell cycle dysregulation is a key feature in lung adenocarcinoma.

### Development of a centrosome relevant prognostic model

**Figure 3:**
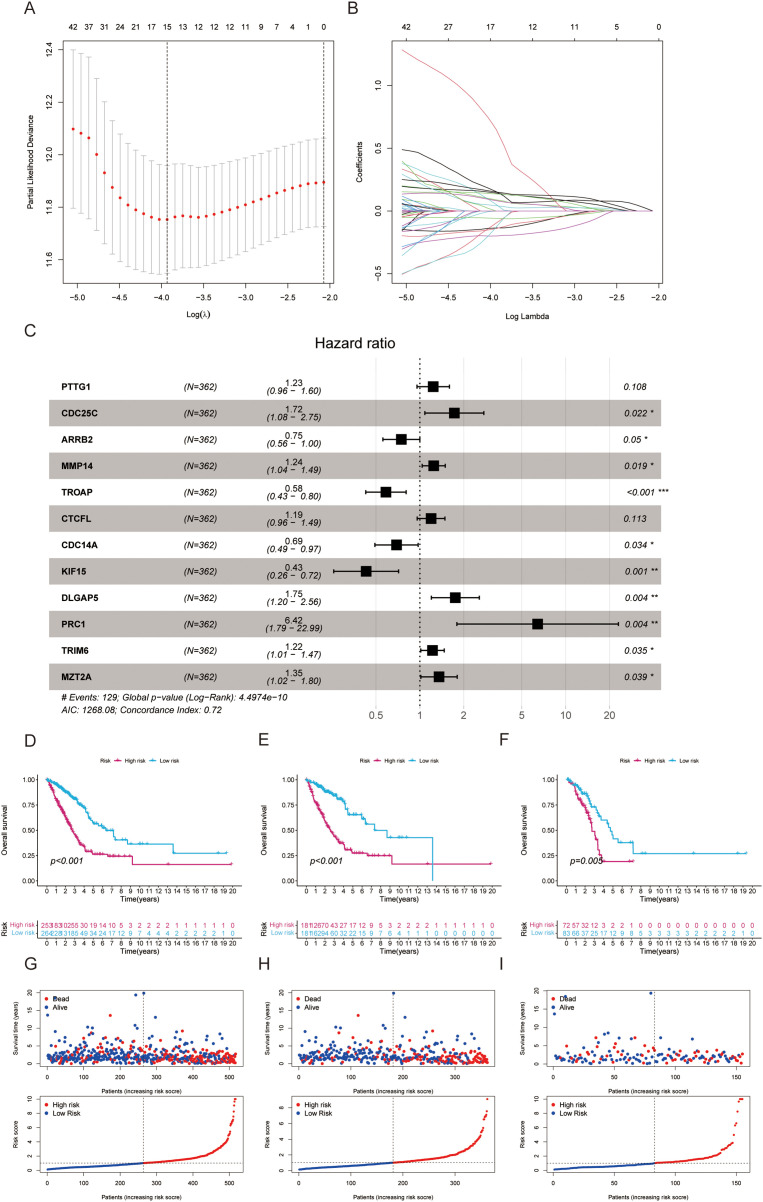
Establishment of a prognostic model based on centrosome-related genes (A) LASSO coefficients for the four genes related to sphingolipid metabolism. (B) Gene discovery to conduct a predictive risk score model. (C) The forest plot illustrates the HR values and *p-values* of the model genes. (D) The survival analysis results for all patients. (E) The survival analysis results for patients in the training set. (F) The survival analysis results for patients in the validation set. (G) The distribution and risk score ranking of all patients. (H) The distribution and risk score ranking of patients in the training set. (I) The distribution and risk score ranking of patients in the validation set. “*” for *p* < 0.05, “**” for *p* < 0.01, and “***” for *p* < 0.001.

**Figure 4:**
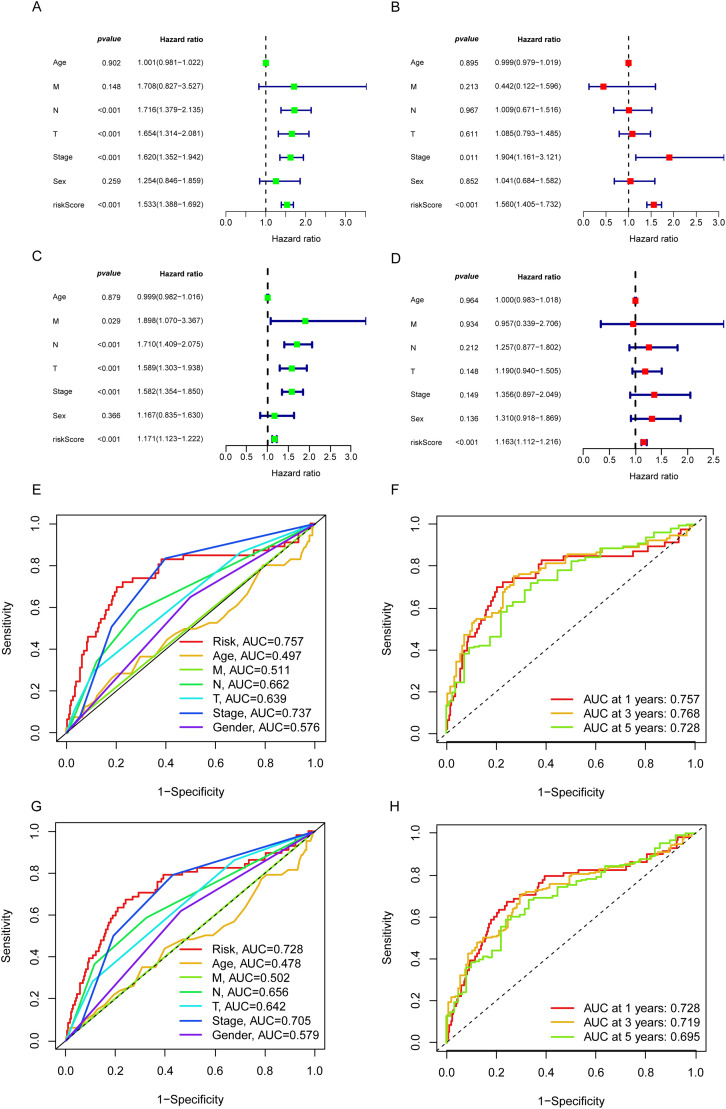
The independent prognostic analysis and model performance evaluation. (A) The forest plot displays the results of the univariate Cox analysis for the influencing factors in the training set. (B) The forest plot displays the results of the multivariate Cox analysis for the influencing factors in the training set. (C) The forest plot displays the results of the univariate Cox analysis for the influencing factors in the validation set. (D) The forest plot illustrates the results of the multivariate Cox analysis for the influencing factors in the validation set. (E) The 5-year ROC curves and corresponding AUC values based on various factors in the training set are presented in the graph. (F) The time-dependent ROC curves and corresponding area under the curve (AUC) values based on the training set are shown in the graph. (G) The 5-year ROC curves and corresponding AUC values based on various factors in the validation set are presented in the graph. (H) The time-dependent ROC curves and corresponding area under the curve (AUC) values based on the validation set are shown in the graph.

From the pool of 726 CAGs, 724 genes had RNA-seq data available (Fig. 2A). Among these, 115 genes showed significant differences between normal and tumor tissues (Fig. 2B), with 76 upregulated and 39 downregulated in tumor tissue (Fig. 2C). Principal component analysis revealed a clear distinction between normal and tumor tissues (Fig. 2D). Functional enrichment analysis on the differentially expressed CAGs showed that they were primarily associated with cell cycle-related processes such as mitotic phase transition and sister chromatid segregation (Fig. 2E). In the KEGG analysis, the most significantly enriched pathway was the cell cycle, alongside others like oocyte meiosis and tumor-related pathways (Fig. 2F), suggesting a dysregulated cell cycle in patients. Univariate Cox regression identified 155 prognosis-related genes (Table S1), and LASSO regression was used to narrow this down (Fig. 3A,B). Multivariate regression analysis highlighted 12 key genes impacting prognosis: PTTG1, CDC25C, ARRB2, MMP14, TROAP, CTCFL, CDC14A, KIF15, DLGAP5, PRC1, TRIM6, and MZT2A (Fig. 3C). Based on these, a predictive model was constructed, with 10 genes serving as independent prognostic factors. Survival analysis divided patients into HRLAs and LRLAs, showing that those in the HRLAs group had significantly poorer outcomes (Fig. 3D–F). This was further validated (Fig. 3G). CAGS was calculated for each sample, allowing risk stratification into HRLAs and LRLAs. The cutoff was based on the mean CAGS in the training cohort, dividing it into LRLAs (n = 181) and HRLAs (n = 181) (Fig. 3H). In the validation cohort, patients were split into LRLAs (n = 83) and HRLAs (n = 72) (Fig. 3I). Expression levels and individual prognostic effects of genes in the model were examined, with seven showing significant prognostic associations (Fig. S1). Except for CTCFL, other genes showed significant expression differences between groups (Fig. S2). In the training cohort, CAGS, T stage, N stage, and stage correlated with overall survival, but in the validation cohort, only CAGS was significantly associated (Fig. 4A–D), likely due to limited tumor staging data. CAGS consistently provided a more accurate survival prediction (Fig. 4E–G). A time-based ROC curve for one, three, and five years showed AUC values of 0.757, 0.768, and 0.728 in the training group, and 0.728, 0.719, and 0.695 in the validation group (Fig. 4F–H), indicating the model’s strong performance and reliability in forecasting LUAD prognosis.

### A nomogram for predicting survival duration

**Figure 5:**
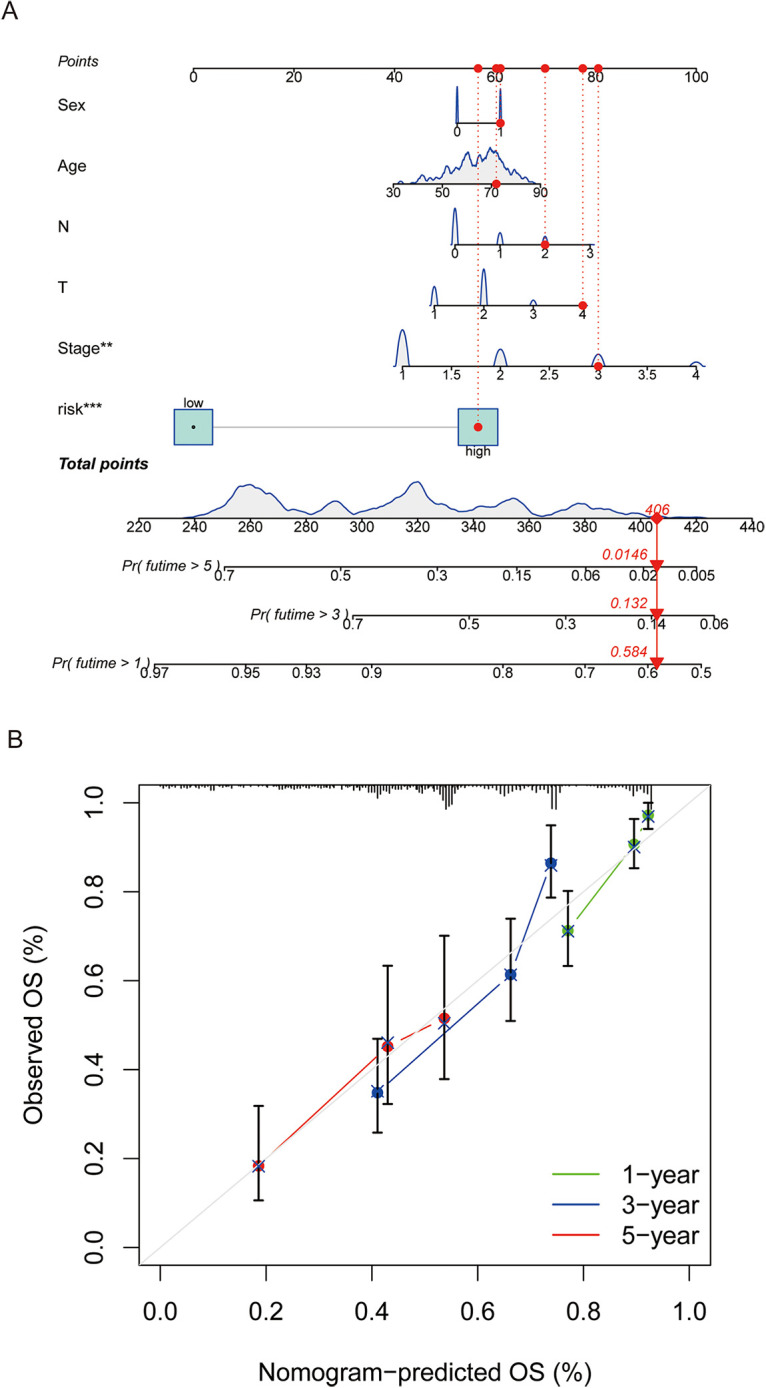
A nomogram was constructed to predict patient prognosis. (A) A nomogram was constructed based on sex, age, tumor stage, and risk score to predict patient prognosis. (B) Calibration curve is used to assess the accuracy and consistency of the prediction results from the nomogram. “**” for *p* < 0.01, and “***” for *p* < 0.001.

We developed a nomogram incorporating factors such as age, sex, stage, and CAGS to evaluate the overall survival of patients (Fig. 5A). Calibration curves at various time points were employed to demonstrate the nomogram’s accuracy in predicting the survival of LUAD patients (Fig. 5B). The analysis results indicate that the nomogram provides an accurate assessment of prognosis, offering valuable guidance for clinical decision-making in the treatment of LUAD.

### Description of tumor mutation landscape

**Figure 6:**
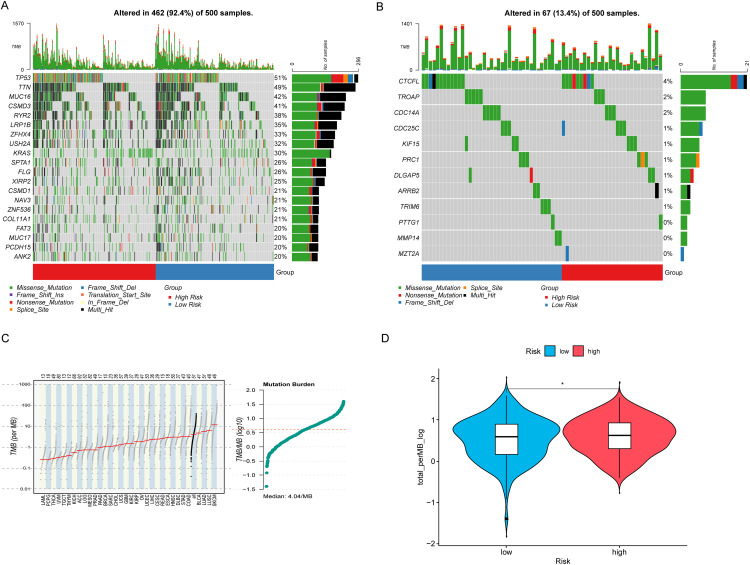
Tumor mutation landscape. (A) The waterfall plot shows the top 20 genes with the highest mutation rates in lung adenocarcinoma along with the nature of their mutations. (B) The waterfall plot illustrates the mutation landscape and the nature of mutations in the model genes in lung adenocarcinoma. (C) The mutation profile across different tumors is generally consistent with the analysis, with LUAD exhibiting a significantly higher tumor mutation burden. (D) LUAD patients with higher risk exhibit a higher tumor mutation burden. “*” for *p* < 0.05.

The description of the tumor mutation landscape provides a comprehensive overview of the overall characteristics and distribution of gene mutations within the tumor. This visual representation of mutation status in tumor samples helps researchers better understand the genetic variation features of the tumor and identify potential therapeutic targets. The results indicate that many of the top 20 CAGs with higher mutation rates are known cancer drivers, with minimal differences observed between HRLAs and LRLAs (Fig. 6A). This suggests that these mutations do not significantly contribute to identifying HRLA patients. For the genes included in our model, mutation rates were found to be very low, with the highest rate being only four percent of patients, and some of these mutations were non-significant (Fig. 6B). This leads us to believe that the normal expression of proteins encoded by these genes likely has a more direct impact on tumor progression and patient prognosis, rather than their mutational status. Additionally, we calculated the tumor mutation burden (TMB) for the samples and found minimal differences between the two groups (Fig. 6C,D), further supporting the notion that protein expression, rather than mutation rates, may be more crucial in influencing disease progression and outcomes in these patients.

### Dissection of differentially expressing CAGs in HRLAs and LRLAs

**Figure 7:**
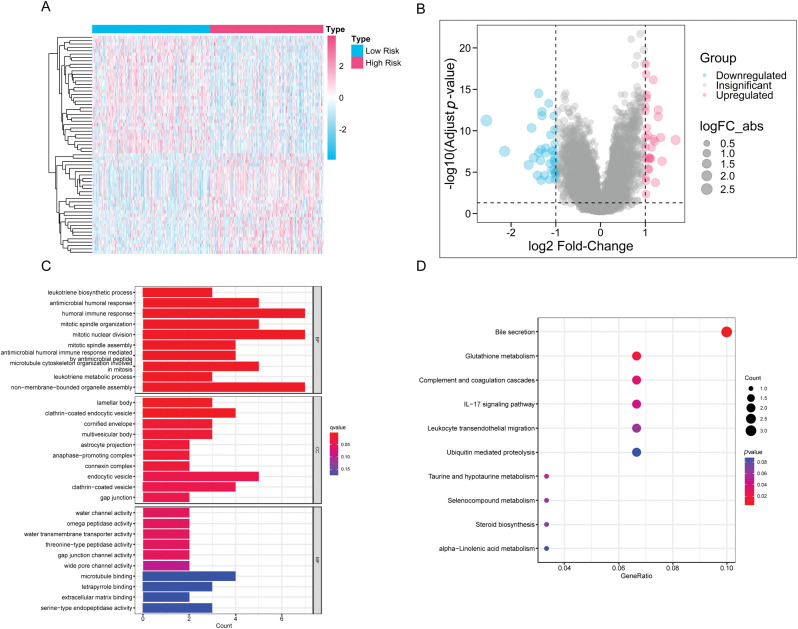
The differences in gene expression and functionality between the HRLAs and LRLAs. (A) The heatmap displays the expression of differentially expressed genes between the two groups. (B) The forest plot displays the upregulation and downregulation of differentially expressed genes and their fold changes. (C) The bar plot presents the results of differentially expressed genes in GO enrichment analysis. (D) The bubble plot displays the results of differentially expressed genes in KEGG enrichment analysis.

By dividing the patients into two groups, we performed differential expression analysis according to the filtering criteria outlined in the methodology. A total of 66 differentially expressed genes were identified (Fig. 7A,B). These genes were then subjected to functional enrichment analysis using KEGG and GO. We highlighted the top 10 significant results from GO enrichment across biological processes (BP), cellular components (CC), and molecular functions (MF). Notable biological processes included antimicrobial humoral response, mitotic spindle organization, and antimicrobial immune response mediated by peptides (Fig. 7C). These findings suggest that immune function and cell cycle regulation are implicated in lung adenocarcinoma progression. In the KEGG pathway analysis, significant enrichment was observed in pathways such as the IL-17 signaling pathway, leukocyte transendothelial migration, and various metabolic pathways (Fig. 7D). These results highlight the importance of immune functionality in the progression of lung adenocarcinoma and serve as a foundation for our continued focus on immune-related processes in subsequent analyses.

### GSVA

**Figure 8:**
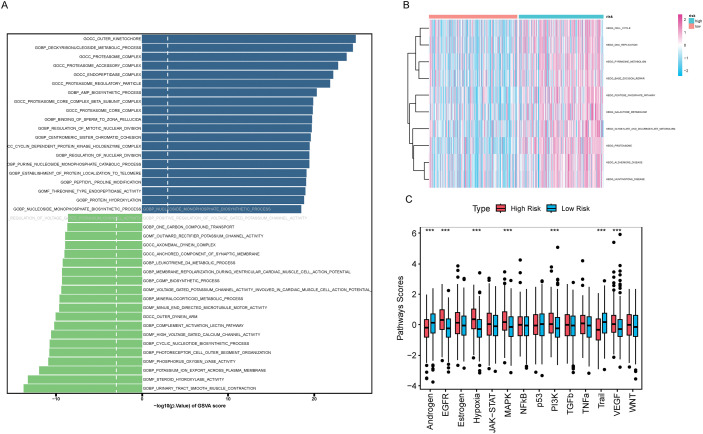

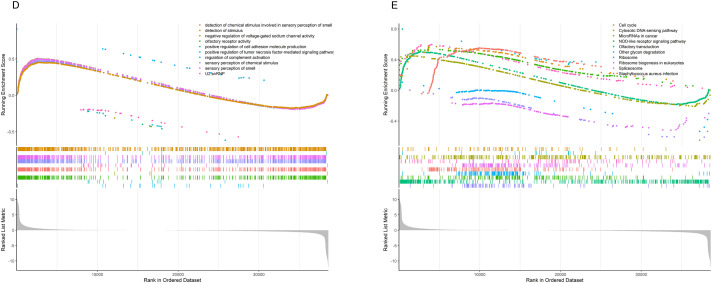
Results of GSVA and GSEA Analyses (A) The bidirectional bar chart presents the differences in GO pathway scores within the samples between the two groups. (B) The heatmap presents the differences in KEGG pathway scores within the samples between the two groups. (C) The differences in the scores of 14 classic tumor pathways between the two groups. (D) The results of Gene Set Enrichment Analysis (GSEA) in the Gene Ontology (GO) standard gene sets. (E) The results of Gene Set Enrichment Analysis (GSEA) in the KEGG standard gene sets. “***” for *p* < 0.001.

We applied GSVA to assess pathway scores for KEGG and GO gene sets across each sample and identified pathways with significant differences between HRLAs and LRLAs. Regardless of the gene set used, we observed notable differences in pathway scores related to the cell cycle and immune-related processes between the two groups (Fig. 8A,B). Additionally, using the Progeny package to score 14 classic tumor pathways revealed significant differences in pathway activity. Specifically, the androgen, EGFR, hypoxia, MAPK, PI3K, Trail, and VEGF pathways showed either increased or decreased activity (Fig. 8C), consistent with previously reported changes in LUAD. Overall, the GSVA analysis confirmed that pathways related to the cell cycle and immune function are disrupted in tumor tissue, contributing to the progression of the disease.

### GSEA

We performed GSEA to evaluate pathway enrichment for upregulated and downregulated genes in tumor tissue, presenting the top 10 most significant pathways for each set (Fig. 8D,E). Special attention was given to immune-related signaling pathways. The results revealed decreased activity in pathways related to humoral immune response, immunoglobulin complex, positive regulation of humoral immune response, positive regulation of innate immune response, regulation of humoral immune response, regulation of innate immune response, T cell chemotaxis, and T cell receptor complex in tumor tissue (Fig. S3). This clear evidence of immune dysfunction in the tumor tissue suggests it may play a critical role in tumor progression, prompting further investigation into immune cell infiltration and the tumor microenvironment.

### Immune infiltration

**Figure 9:**
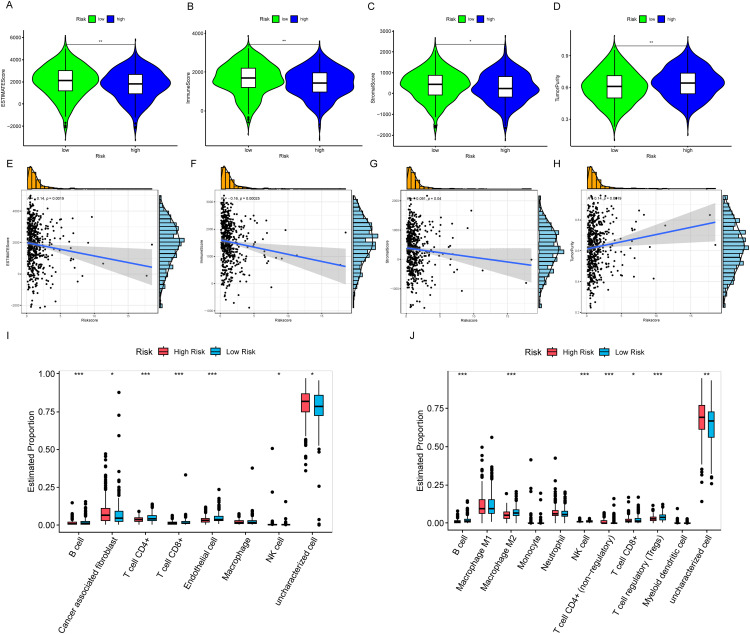
Characterization of the immune microenvironment (A) ESTIMATE score is lower in the HRLAs group. (B) Immune score is lower in the HRLAs group. (C) Stromal score is lower in the HRLAs group. (D) Tumor purity is higher in the HRLAs group. (E) Riskscore is significantly negatively correlated with ESTIMATE score. (F) Riskscore is significantly negatively correlated with immune score. (G) Riskscore is significantly negatively correlated with stromal score. (H) Riskscore is significantly positively correlated with tumor purity. (I) Epic immunoinfiltration analysis displayed the percentage content and differences of various types of cells. (J) QuanTIseq immune infiltration analysis showed the percentage content and differences of various types of cells. “*” for *p* < 0.05, “**” for *p* < 0.01, and “***” for *p* < 0.001.

First, we assessed the samples using ESTIMATE, and the results showed that HRLAs had lower ESTIMATE scores, immune scores, and stromal scores, while exhibiting higher tumor purity (Fig. 9A–D). Using the Spearman method, we examined the correlation between CAGS and these four scores. As hypothesized, increasing risk scores were associated with decreasing ESTIMATE, immune, and stromal scores, and increasing tumor purity (Fig. 9E–H). All *p-values* were below 0.05, confirming statistical significance. This suggests that HRLAs exhibit immune dysfunction and are in a relatively immunosuppressed state. Next, we employed six different methods to evaluate immune cell infiltration in tumor tissues. As described in the methods, two of these methods calculated the absolute proportions of immune cells, revealing significant differences in immune cell infiltration between the two groups (Fig. 9I,J). Similar results were obtained using the other four methods (Fig. S4). Immune cells such as B cells, CD4+ T cells, and CD8+ T cells were less abundant in the HRLAs group, likely due to their cytotoxic effects on tumor cells. Conversely, certain immune cells might promote immune evasion by the tumor, facilitating its progression. Thus far, we have confirmed that patients identified as high risk, based on centrosome-related factors, exhibit immune dysregulation. This prompted us to conduct drug susceptibility analysis and assess the potential response to immunotherapy to explore promising treatment approaches.

### Drug sensitivity and immunotherapy

**Figure 10:**
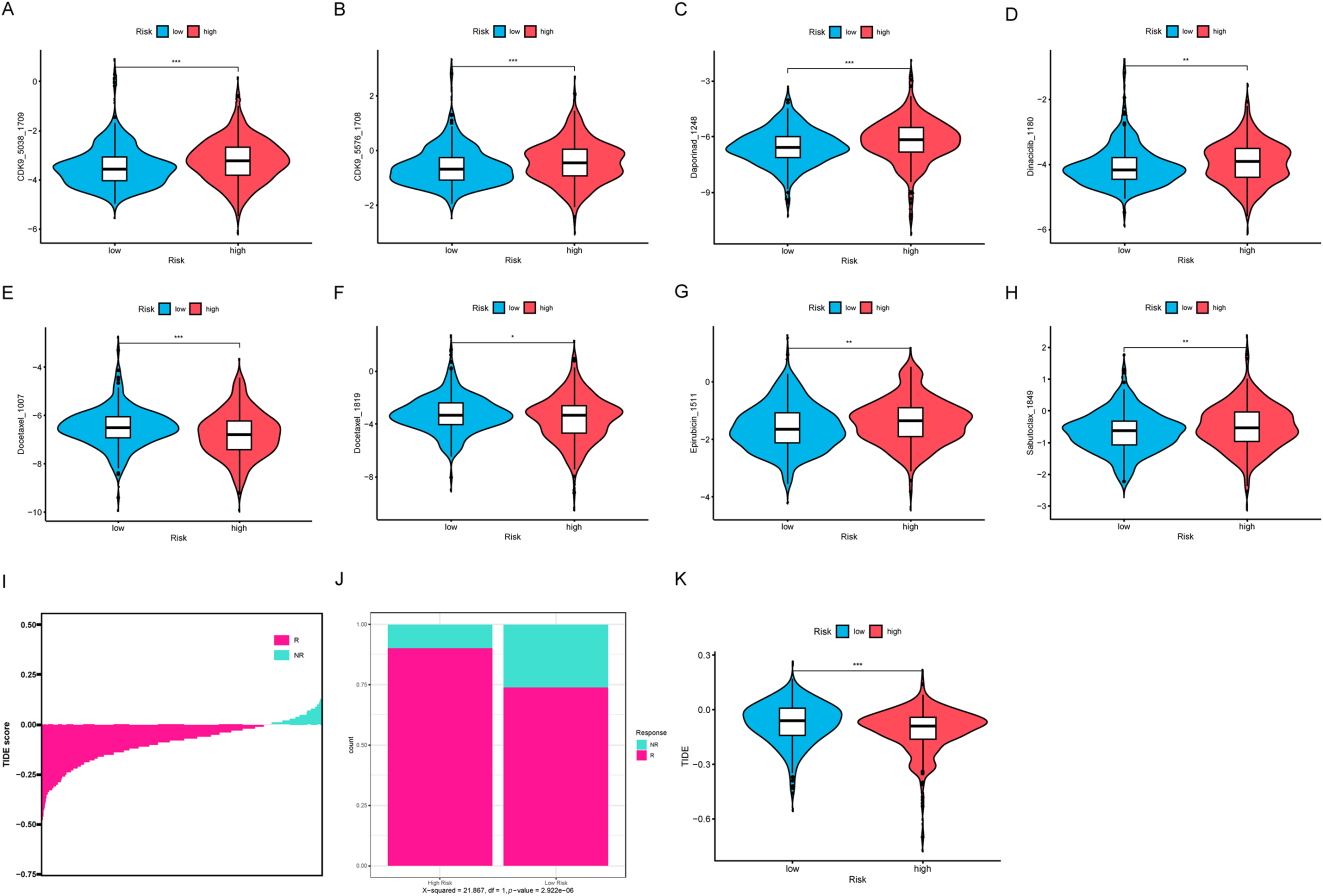
Drug sensitivity and immune therapy response (A–H) The presentation includes 8 potential effective drugs and compares the differences in their IC50 values between the two groups. (I) The samples’ TIDE score ranking, indicating their response to immunotherapy. (J) The proportion of patients in the HRLAs and LRLAs who respond to immunotherapy. (K) The difference in TIDE scores between the two groups. “*” for *p* < 0.05, “**” for *p* < 0.01, and “***” for *p* < 0.001.

We analyzed the sensitivity of 198 commonly used drugs in tumor samples and calculated their IC50 values. Drugs with a mean IC50 value less than 1 in tumor samples were considered significantly effective chemotherapy agents. Out of the 198 drugs, 8 met this criterion (Fig. 10A–H). Among these, we found that only two compounds, including docetaxel, had lower IC50 values in the HRLAs group, indicating that docetaxel may be more effective for HRLAs patients (Fig. 10E,F). This suggests that docetaxel could be a suitable chemotherapy option for HRLAs patients identified based on centrosome-related gene profiles. Additionally, we used the TIDE database to predict the response of these samples to immunotherapy (Fig. 10I). The TIDE score reflects the ability of immune evasion, with higher scores indicating stronger immune evasion and a poorer response to immunotherapy. Our results showed that a larger proportion of HRLAs patients are likely to respond to immunotherapy, significantly more than those in the LRLAs group (Fig. 10J). Moreover, HRLAs patients had lower TIDE scores, suggesting they are more sensitive to immunotherapy (Fig. 10K). These findings indicate that immunotherapy could be an effective treatment for HRLAs patients, and the results provide valuable guidance for clinicians in selecting appropriate therapies for these patients based on centrosome-related gene profiles.

### Immunohistochemistry

**Figure 11:**
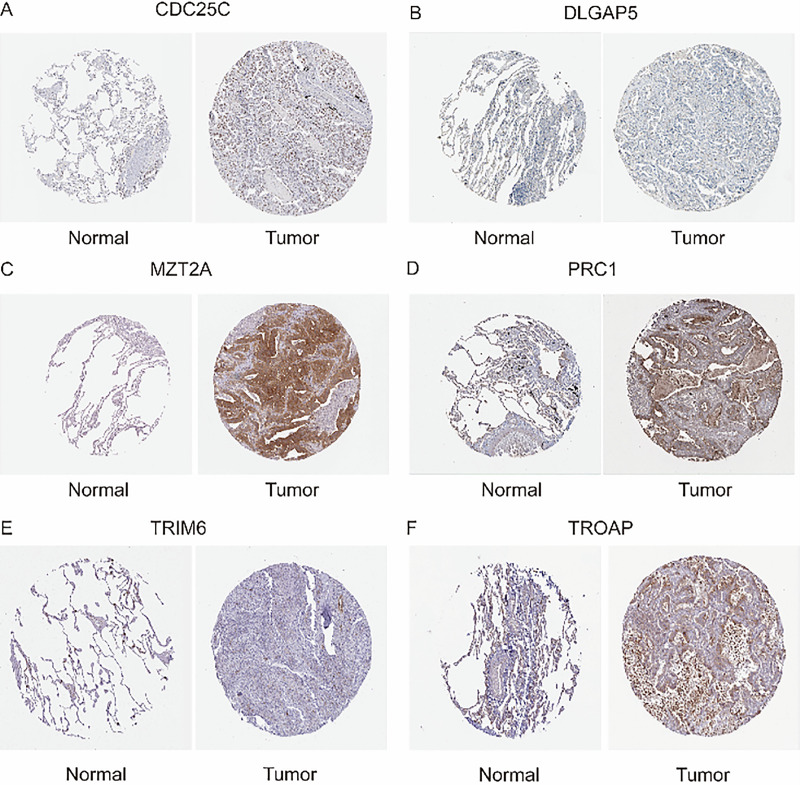
Immunohistochemistry (A) Expression of CDC25C in Normal Lung Tissue and Lung Adenocarcinoma Tissue. (B) Expression of DLGAP5 in Normal Lung Tissue and Lung Adenocarcinoma Tissue. (C) Expression of MZT2A in Normal Lung Tissue and Lung Adenocarcinoma Tissue. (D) Expression of PRC1 in Normal Lung Tissue and Lung Adenocarcinoma Tissue. (E) Expression of TRIM6 in Normal Lung Tissue and Lung Adenocarcinoma Tissue. (F) Expression of TROAP in Normal Lung Tissue and Lung Adenocarcinoma Tissue.

To validate the reliability of our previous bioinformatics analyses, we obtained immunohistochemistry data for six genes from the Human Protein Atlas (Fig. 11A–F). These genes, identified in our model as independent predictors of patient prognosis, were shown to significantly influence survival outcomes based on their expression levels. We compared their expression in both normal tissue and lung adenocarcinoma tissue. The findings demonstrated that the expression profiles of these model genes in tumor tissues are consistent with their predicted impact on prognosis. Specifically, genes that promote tumor progression and worsen prognosis were found to be highly expressed in lung adenocarcinoma tissues compared to normal tissues. Therefore, our analysis, based on RNA-seq results from lung adenocarcinoma, is robust and reliable.

### Knocking down PRC1 inhibits the proliferative capacity of A549 cells

**Figure 12:**
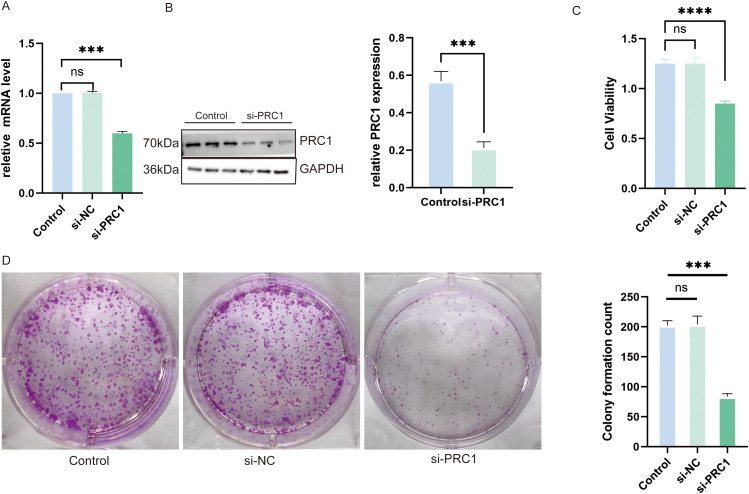
Knocking down PRC1 significantly inhibits the viability and proliferation of A549 cells (A) The qRT-PCR results show that si-RNA successfully reduced the mRNA levels of PRC1 (n = 3). (B) Western blot results show that si-RNA treatment reduced the protein levels of PRC1 in the cells (n = 3). (C) The CCK-8 results show that knocking down PRC1 significantly inhibits the viability of A549 cells (n = 3). (D) The results from the plate cloning assay show that after knocking down PRC1, there is a reduction in cell colony formation, indicating a decrease in cell proliferation ability (n = 3). “***” for *p* < 0.001, “****” for *p* < 0.0001 and “ns” for no statistical difference.

We selected the PRC1 gene, which has the highest hazard ratio (HR) in the model and is a strong independent predictor of patient prognosis, for further validation in vitro. Using qRT-PCR, we confirmed that siRNA successfully reduced PRC1 mRNA levels (Fig. 12A). Western blot analysis further validated that PRC1 was effectively knocked down, both at the mRNA level and post-translationally (Fig. 12B). The results of the CCK-8 assay showed that silencing PRC1 significantly inhibited the proliferation of A549 cells, indicating that the protein encoded by PRC1 plays a critical role in the proliferation process of A549 cells (Fig. 12C). Additionally, the plate cloning experiment demonstrated that after siRNA treatment, colony formation was notably reduced, further supporting the essential role of PRC1 in promoting cell proliferation (Fig. 12D). These findings collectively indicate that PRC1 is a key factor influencing cellular proliferation and may serve as an important biomarker for predicting patient prognosis.

### Knocking down PRC1 significantly inhibits the migration and invasion abilities of A549

**Figure 13:**
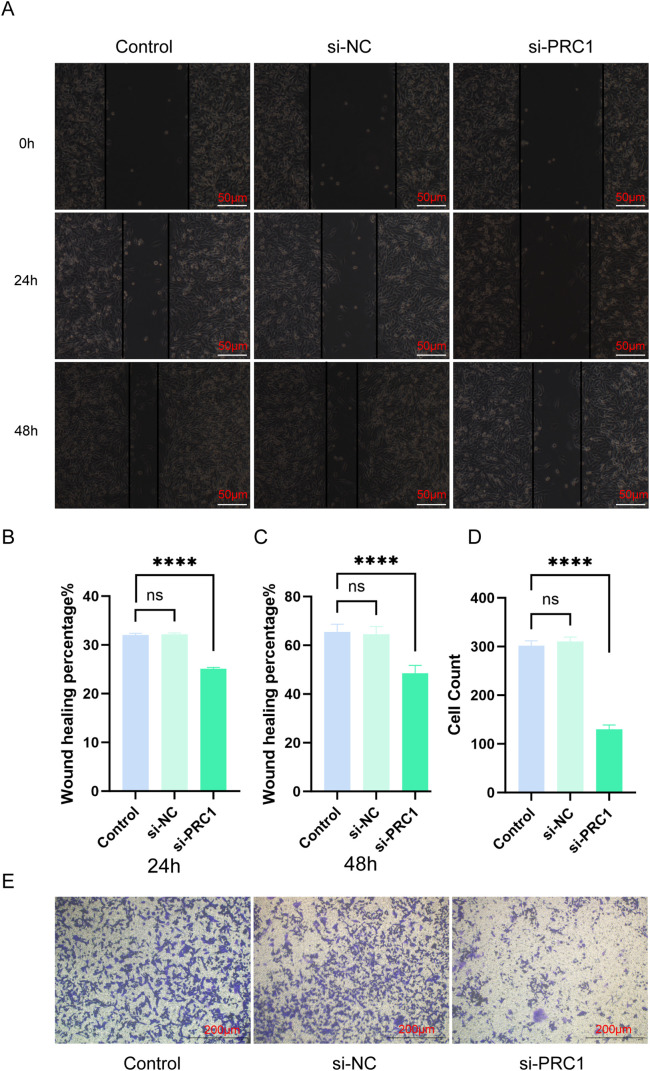
Knocking down PRC1 inhibits the migration and invasion abilities of A549 cells (A) The scratch assay results show that knocking down PRC1 weakens the migration ability of A549 cells (n = 3). (B) Statistical analysis of the healed area at 24 h (n = 3). (C) Statistical analysis of the healed area at 48 h (n = 3). (D) Statistical results of cell counting in Transwell experiments (n = 3). (E) The results of the Transwell experiment show that knocking down PRC1 significantly reduces the number of cells invading through the small chambers (n = 3). “****” for *p* < 0.0001 and “ns” for no statistical difference.

We evaluated the effect of PRC1 knockdown on the migration and invasion abilities of A549 cells using scratch assays and Transwell experiments. In the scratch assay, knocking down PRC1 significantly reduced the wound-closing ability of A549 cells compared to the control group, indicating a marked decrease in cell migration, as the cells were less effective in moving towards the wound area (Fig. 13A–C). Similarly, in the Transwell assay, the number of A549 cells passing through the membrane to the lower chamber was significantly lower in the PRC1 knockdown group than in the control group (Fig. 13D,E). This reduction in cell migration through the membrane pores indicates a significant inhibition of both migration and invasion capabilities. Together, these results strongly suggest that PRC1 plays a critical role in promoting migration and invasion in A549 cells, highlighting its importance in cell motility and invasive processes.

## Discussion

Lung adenocarcinoma (LUAD) is one of the most common forms of lung cancer, primarily affecting adults [25]. Despite therapeutic advancements, the 5-year survival rate for LUAD patients has shown only modest improvement [26]. Thus, developing risk-based stratification strategies and personalized treatment regimens for LUAD is critical. In this study, we focused on centrosome-associated genes (CAGs). Our analysis of immune cells revealed that patients with poor prognoses exhibited an unfavorable immune profile, characterized by low immune scores, low ESTIMATE scores, and high tumor purity, compared to those with better outcomes. Functional analysis demonstrated a correlation between CAG expression, immune function, and the cell cycle. Furthermore, a prognostic risk model based on CAGs accurately predicted LUAD patient outcomes. These findings may contribute to the development of precision therapies for LUAD and assist clinicians in determining effective treatment plans.

Our prognostic model, built on CAGs, reliably predicts patient risk and outcomes. This model was used to develop a nomogram for personalized treatment support and clinical guidance. Among the 12 genes in our model, six were identified as independent prognostic factors for LUAD, with their expression directly affecting patient prognosis. CDC25C, a key regulator of cell division, dephosphorylates cyclin B-bound CDC2 to initiate mitosis and inhibits p53-induced growth arrest. DLGAP5 is involved in centrosome localization, kinetochore assembly, and mitotic spindle organization, facilitating microtubule binding. MZT2A plays a crucial role in cytokinesis, peaking during the S and G2/M phases of mitosis. PRC1 is a key factor in cytokinesis, showing high expression during mitosis and associating with the mitotic spindle. TRIM6, though its function is unclear, is part of the TRIM gene family and may play a role in immune regulation. TROAP is associated with cell adhesion and is located in the cytoplasm. These genes, primarily involved in cell cycle regulation, are implicated in tumor initiation and progression. Notable differences were observed in cell cycle-related pathways between normal lung tissue and lung adenocarcinoma, as well as between high-risk (HRLAs) and low-risk (LRLAs) patient groups. These analyses also revealed immune dysfunction, prompting further exploration of immune infiltration.

The tumor immune microenvironment (TIME) plays a significant role in patient prognosis, as tumor progression is closely linked to changes in the surrounding stroma and immune cells [27]. The ESTIMATE algorithm predicts tumor purity based on gene profiles and the proportion of immune and stromal cells in the tumor [28]. Lower immune scores and higher tumor purity have been associated with poorer prognosis [29]. In our study, the ESTIMATE algorithm confirmed that patients with better prognoses had higher immune scores and lower tumor purity. Immune cell infiltration analysis also revealed a decrease in almost all immune cells in the tumor tissues of HRLAs patients, indicating that impaired immune function contributes to disease progression. We then focused on identifying potential drug targets and evaluating immunotherapy effectiveness. Based on CAGs, we identified docetaxel as a promising treatment for HRLAs. Docetaxel, a taxane drug, inhibits microtubule function in tumor cells, preventing their division and growth, and is used to treat various cancers, including non-small cell lung cancer [30–35]. This suggests that docetaxel can be tailored for HRLAs patients to provide personalized care. Additionally, our model predicted that most HRLAs patients would respond well to immunotherapy, making it a viable treatment option to suppress tumor progression and improve prognosis.

To validate the accuracy of our model, we selected PRC1, the gene with the highest CAG score, for *in vitro* validation. Knockdown of PRC1 in A549 cells using siRNA confirmed its role in neoplasm activity and progression. Silencing PRC1 significantly inhibited the vitality, proliferation, migration, and invasion of A549 cells. Since these processes are critical to cancer progression, this supports the reliability of our model and its ability to predict patient prognosis and suggest targeted treatment strategies.

Despite these promising findings, our study has limitations. The lack of clinical specimens prevented us from validating patient sensitivity to drugs and immunotherapy. This will be explored further in future research when conditions allow.

## Conclusion

In this study, LUAD patients were classified into HRLAs and LRLAs based on the correlation between CAGs and overall survival (OS). Immunological and functional analyses revealed that impaired cell cycle regulation disrupts immune system function, contributing to poor prognosis in LUAD patients. These findings lay the groundwork for developing targeted personalized therapies and support the use of risk-based classification for optimizing treatment strategies in LUAD patients.

## Supplementary Materials

Figure S1 The prognostic impact of the expression of the 12 model genes on lung adenocarcinoma patients.

Figure S2 Expression profile of model genes. (A) The heatmap illustrates the expression and inter-group differences of model genes in patients from the training set. (B) The heatmap illustrates the expression and inter-group differences of model genes in patients from the validation set. (C) The heatmap illustrates the expression and inter-group differences of model genes in all patients. (D) Differences and significance of model gene expression between HRLAs and LRLAs. *p<0.05, **p<0.01, ***p<0.001.

Figure S3 GSEA analysis results for 8 immune-related pathways.

Figure S4 Results of 4 immune infiltration analyses. (A) Results of XCELL immune infiltration analysis and the differences in infiltration between HRLAs and LRLAs. (B) Results of CIBERSORT immune infiltration analysis and the differences in infiltration between HRLAs and LRLAs. (C) Results of TIMER immune infiltration analysis and the differences in infiltration between HRLAs and LRLAs. (D) Results of MCPcounter immune infiltration analysis and the differences in infiltration between HRLAs and LRLAs.

## Data Availability

The data used in this study are available at https://xenabrowser.net/ (accessed on 04 December 2024).

## Supplementary Materials

The supplementary material is available online at https://doi.org/10.32604/or.2025.056176.
