# Supplementary figures and images for: Developing a prognostic signature and characterizing the tumor microenvironment based on centrosome-related genes in lung adenocarcinoma
Source: Oncol Res. 2025 Jun 26;33(7):1649–66. doi: 10.32604/or.2025.056176 (PMC12215551; doi:10.32604/or.2025.056176)

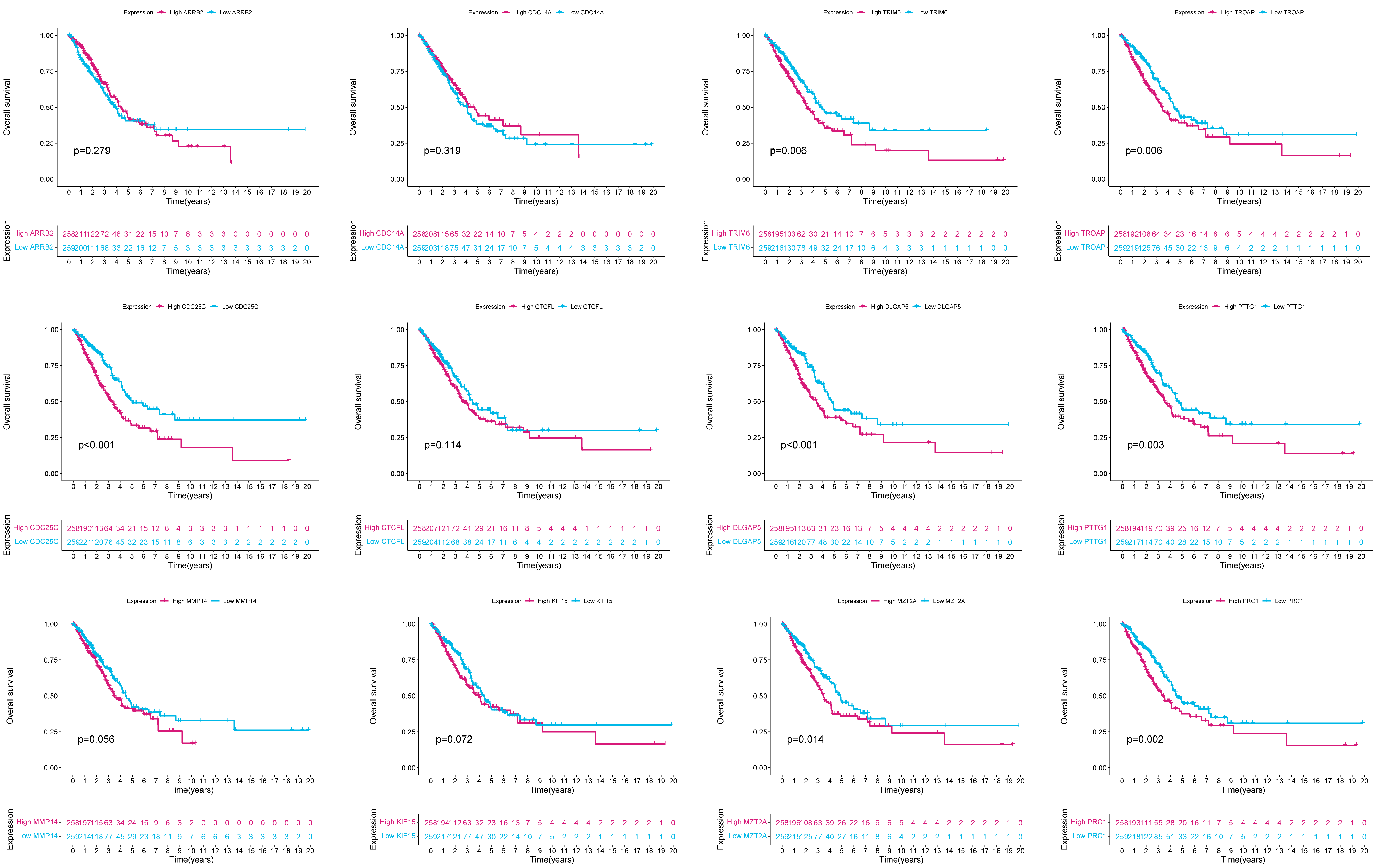

Supplement: Figure S1 [file OncolRes-33-56176-s001.tif]

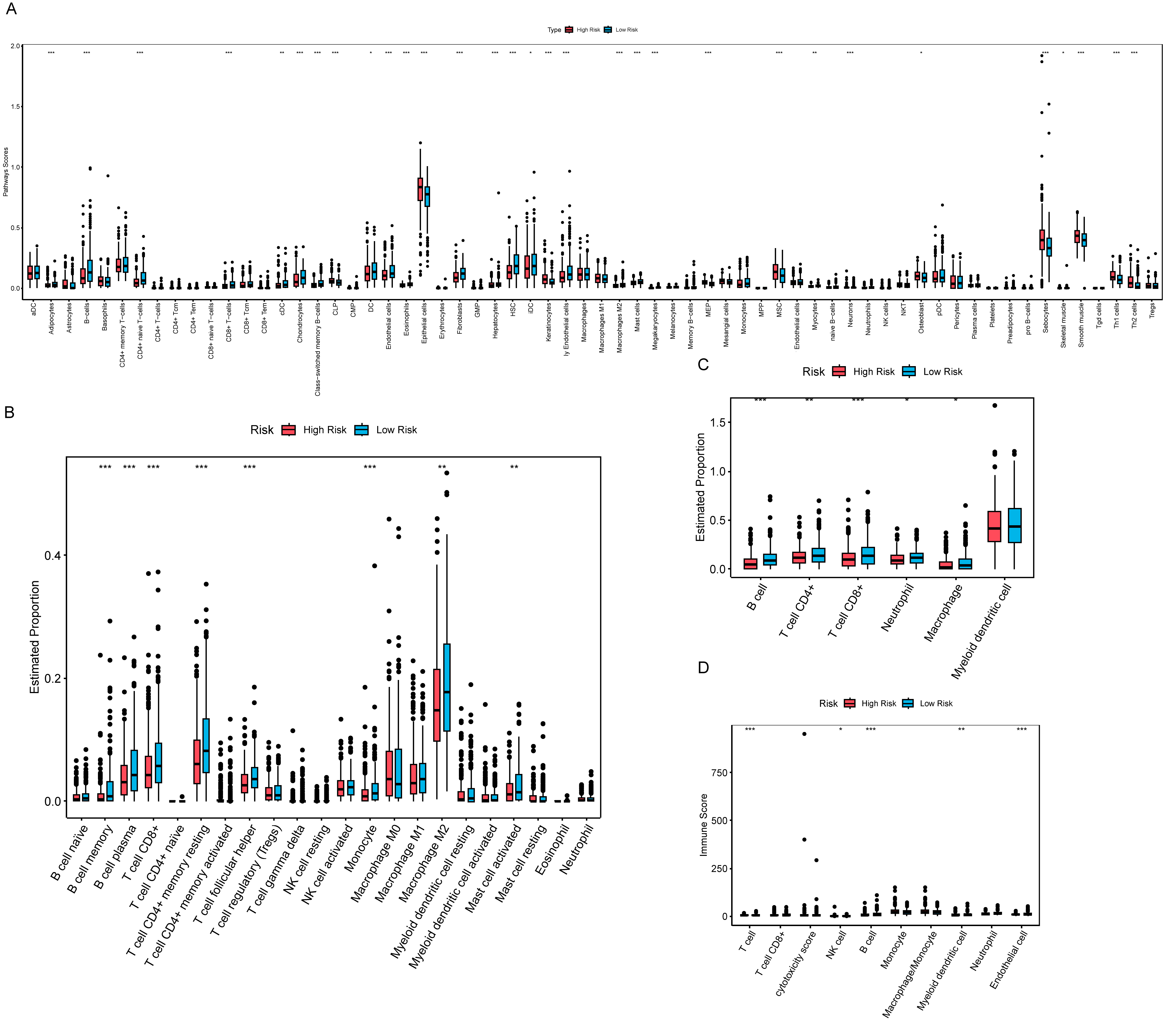

Supplement: Figure S2 [file OncolRes-33-56176-s002.tif]

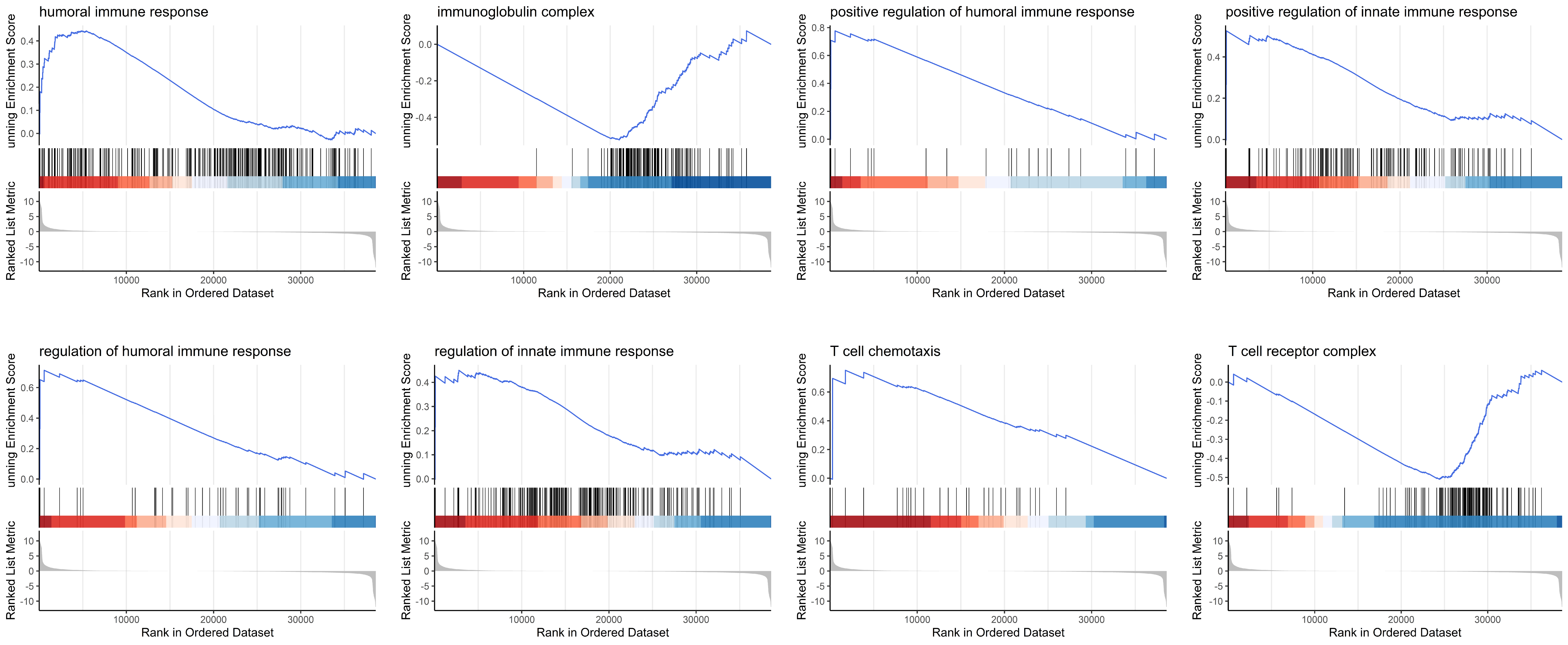

Supplement: Figure S3 [file OncolRes-33-56176-s003.tif]

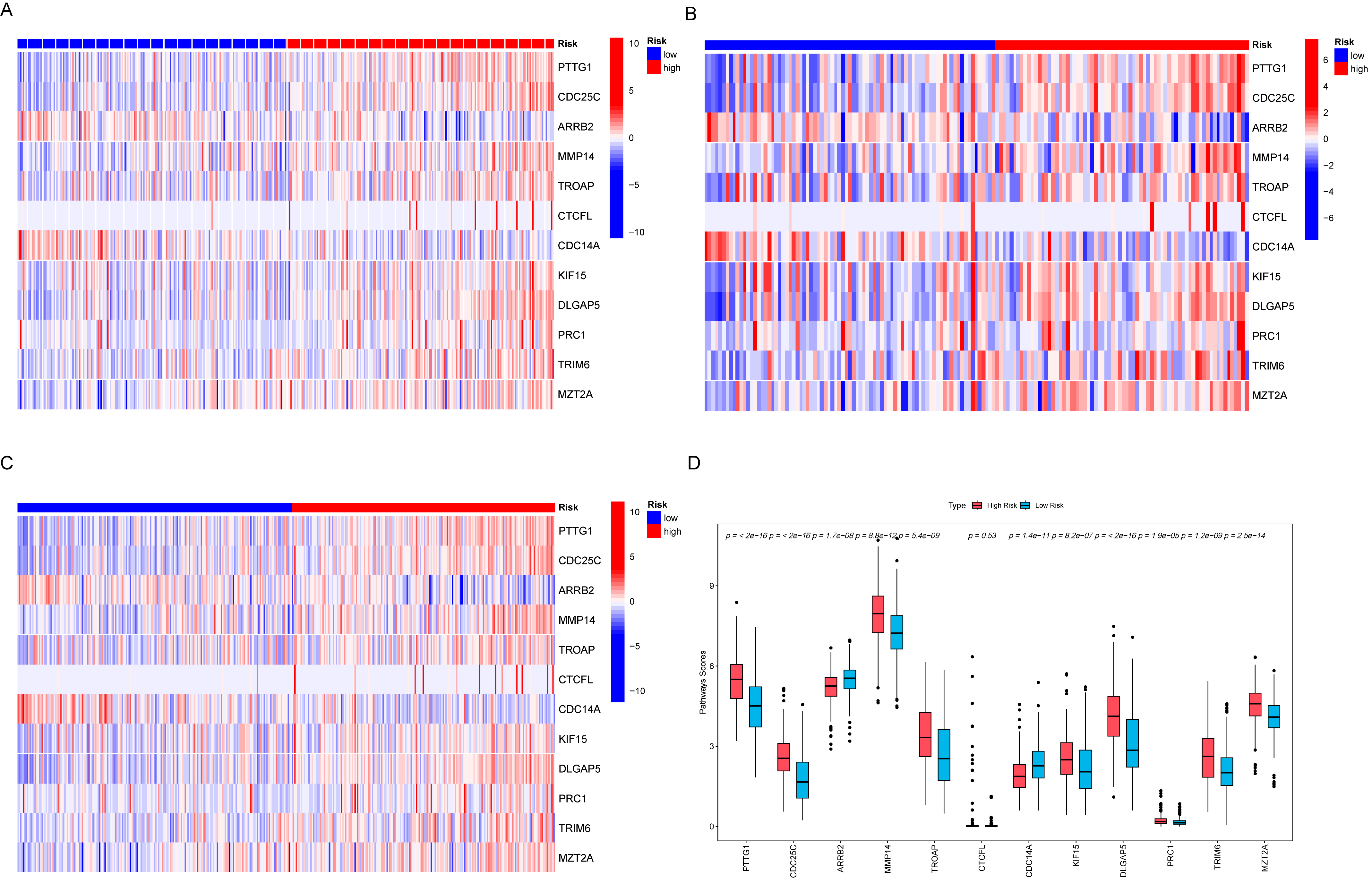

Supplement: Figure S4 [file OncolRes-33-56176-s004.tif]
